# Developing Diagnostic and Therapeutic Approaches to Bacterial Infections for a New Era: Implications of Globalization

**DOI:** 10.3390/antibiotics9120916

**Published:** 2020-12-16

**Authors:** Lucía Fernández, María Dolores Cima-Cabal, Ana Catarina Duarte, Ana Rodriguez, Pilar García, María del Mar García-Suárez

**Affiliations:** 1 Instituto de Productos Lácteos de Asturias (IPLA-CSIC), Paseo Río Linares s/n—Villaviciosa, 33300 Asturias, Spain; lucia.fernandez@ipla.csic.es (L.F.); catarina.leal@ipla.csic.es (A.C.D.); anarguez@ipla.csic.es (A.R.); pgarcia@ipla.csic.es (P.G.); 2 DairySafe Group, Instituto de Investigación Sanitaria del Principado de Asturias (ISPA), Avenida Hospital Universitario s/n, 33011 Oviedo, Spain; 3 Escuela Superior de Ingeniería y Tecnología (ESIT), Universidad Internacional de la Rioja, UNIR. Av. de la Paz, 137, 26006 Logroño, Spain; dolores.cima@unir.net

**Keywords:** infectious diseases, antibiotic resistance, diagnostic systems, new therapies, globalization

## Abstract

In just a few months, the current coronavirus pandemic has exposed the need for a more global approach to human health. Indeed, the quick spread of infectious diseases and their unpredictable consequences, in terms of human lives and economic losses, will require a change in our strategy, both at the clinical and the research level. Ultimately, we should be ready to fight against infectious diseases affecting a huge number of people in different parts of the world. This new scenario will require rapid, inexpensive diagnostic systems, applicable anywhere in the world and, preferably, without the need for specialized personnel. Also, treatments for these diseases must be versatile, easily scalable, cheap, and easy to apply. All this will only be possible with joint support of the governments, which will have to make the requirements for the approval of new therapies more flexible. Meanwhile, the pharmaceutical sector must commit to prioritizing products of global interest over the most profitable ones. Extreme circumstances demand a vehement response, and any profit losses may well pay dividends going forward. Here, we summarize the developing technologies destined to face the current and future health challenges derived from infectious diseases and discuss which ones have more possibilities of being implemented.

## 1. Introduction

Nowadays, nobody questions the existence of a link between globalization and human health, especially infectious diseases [1]. The prevalence, propagation rate, geographic spread and control of communicable diseases are clearly conditioned by different environmental, demographic, technological and economic factors. These, in turn, are changing as a result of our increasingly globalized world. For instance, global environmental change has a major impact on the incidence and behavior of infectious diseases (https://www.who.int/globalchange/environment/en/). Indeed, the growing threats to global water supplies caused by climate change may affect the vulnerability of some populations to infections. Similarly, demographic changes, such as population mobility and the increase in urbanization, may accelerate the spread of pathogens to new areas. In addition, it is important to consider the differences in vulnerability to infectious diseases between developed and developing countries. Changes in the world economy, especially those concerning global trade, have an impact on infectious diseases that goes beyond the simple exchange of goods and services, as they too influence social welfare and access to a proper health care system. Generally, globalization leads to greater inequalities between countries, including in the access to technological and medical advances. However, public health systems need to be ready to provide an efficient sanitary response to both endemic and pandemic diseases. Addressing this challenge will involve the development of improved methods for prevention, control and treatment of these infections [1]. These new techniques will then have to reach countries all over the world in order to effectively alleviate the global infection burden.

Globalization also plays a role in the spread of emerging (new unknown diseases) and re-emerging infectious diseases (endemic diseases with antibiotic resistance), which represent a public health threat with serious social, political, and economic consequences. Some of the most significant emerging and re-emerging infectious agents over the last decade have included Ebola virus (Africa); Middle East respiratory syndrome coronavirus or MERS (Middle East); Zika, chikungunya, yellow fever and dengue viruses (North and South America); and severe acute respiratory syndrome *coronavirus* 2 or SARS-CoV-2 (worldwide). However, the most striking infections [2] might divert the attention of public health authorities from other lethal infectious diseases with epidemic potential. In developed countries, emerging infections are adding to the risks associated with an aging population affected by chronic diseases and more susceptible to infectious diseases. By contrast, in developing countries, infectious diseases add to problems like limited financial resources, malnutrition and poorly controlled use of antimicrobial drugs. To make the scenario worse, drug resistance is emerging in many bacterial pathogens, such as *Acinetobacter baumannii*, *Pseudomonas aeruginosa*, Enterobacteriaceae, *Enterococcus faecium*, *Staphylococcus aureus*, *Helicobacter pylori*, *Campylobacter* spp., Salmonellae, *Neisseria gonorrhoeae*, *Streptococcus pneumoniae*, *Haemophilus influenza* and *Shigella* spp. Indeed, antimicrobial resistance (AMR) is one of the main challenges of global concern for human health. For example, multidrug resistance has been estimated to cause about 29,000 deaths in the United States each year, leading to a health care cost of more than $4.7 billion [3]. In Europe, the estimate is over 33,000 deaths, with a cost of $1.5 billion in direct and indirect costs [4]. Nonetheless, the biggest problem occurs in developing countries, in which infectious diseases (including gastrointestinal, respiratory, sexually transmitted, and nosocomial infections) remain the leading cause of illness and death. Moreover, the average income in these countries is very low, thereby limiting access to expensive therapies. In these countries, antimicrobial resistance is likely related to inappropriate antibiotic prescription together with limited diagnostic facilities, inadequate patient education, unregulated sale of antimicrobials, and widespread non-human use of these substances, among others [5].

Recent advances in basic scientific research, together with the development of molecular biology techniques, have not only improved infectious disease diagnosis, but have also provided relevant data about pathogenesis and epidemiology. As such, science offers the necessary tools for appropriate disease prevention and control. However, the progress made in this field over the past century is now facing a new challenge. The available techniques for disease detection and treatment must be adapted to deal with global health problems. In response to this challenge, several molecular assays have been developed to address pathogen detection and quantitation with high sensitivity and specificity. These include, for instance, some nucleic acid-based detection methods that exhibit a high sensitivity, specificity, accuracy, efficiency, and versatility [6].

Another aspect to consider is that the rising antimicrobial resistance and the heightened risk of viral pandemics might contribute to the increased incidence of bacterial infections. For example, the scarce data available to date show that some coronavirus patients (1% to 10%) contract secondary bacterial infections [7]. On the other hand, the increase in hygiene practices may, at least initially, limit the spread of different microbes, including antibiotic resistant pathogens. However, a more frequent use of biocides may favor antibiotic resistance selection in the long-term due to cross-resistance. The antimicrobial resistance crisis has been further complicated by the dearth in the development and commercialization of novel antibiotics. For instance, only 11 new antimicrobials have been approved by the U.S. Food and Drug Administration (U.S. FDA) since 2017 [8]. In this scenario, it will be essential to promote global initiatives aimed at delivering new compounds that can effectively substitute or complement the current therapeutics.

Overall, to break the vicious circle of resistance, it will be necessary to implement enhanced stewardship policies and design novel, effective antimicrobials. Also, disease risk assessment will help to identify which areas require a greater effort from the public health system to minimize the weakness of the population in the near future. Here, we present a comprehensive review of the research intended to palliate the upcoming risks associated with bacterial infectious diseases, especially those derived from antibiotic resistance. In addition, we discuss which approaches have a greater chance of reaching clinical application.

## 2. State-of-the-Art of Diagnostic Tests for Bacterial Pathogens

**Figure 1:**
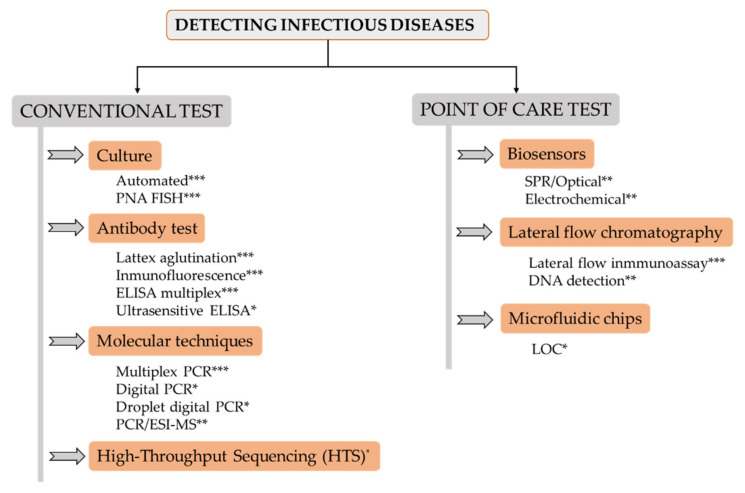
Overview scheme of main pathogen detection systems. Level of implementation in clinical laboratories: * (low); ** (medium); *** (high).

A change in the spread scenario of bacterial infections has become apparent in the present millennium. In the past, wars and poverty were a breeding ground for infectious diseases, caused by bacteria, viruses, parasites and fungi, which sometimes led to millions of deaths in a short period of time. Later on, communicable illnesses stopped being a major threat in developed countries, remaining the main cause of death in developing nations. However, over the last few decades, serious outbreaks have also emerged in developed countries. Some notable examples include Bovine spongiform encephalopathy (BSE), commonly known as “mad cow disease”, Legionnaires’ disease, *E. coli* O157:H7, etc. More recently, the COVID-19 pandemic has completely altered our lives and, consequently, changed our perception of all aspects related to diagnosis and treatment of infectious diseases. Indeed, we are now more aware of the many ways in which good diagnostic strategies can help to fight these illnesses. For example, accurate diagnosis can improve the effectiveness of treatments, help to avoid long-term complications, prevent transmission to others, contribute to the proper use of antibiotics, and even help to stop an outbreak. Still, diagnostic tests have to be adapted to the present needs derived from the emergence of new infectious diseases. This may be achieved by exploiting the latest technologies to develop tests that allow a quick and accurate diagnosis, while being easy to implement anywhere in the world. Some of the main technologies that are currently being used in the clinic or are under development include the following (Figure 1):

### 2.1. Pathogen Culture

Although important progress has been made in the detection and identification of bacteria in the last decade, positive aerobic or anaerobic culture of a potentially sterile fluid (blood, urine, etc.) continues to be the gold standard for diagnosis of most infectious diseases. In some cases, this technique has the disadvantage of taking days and having low sensitivity. Moreover, false-positive cultures, the slow growth of fastidious bacteria or the potential growth of commensal microorganisms can lead to unnecessary antibiotic use. Usually, the Gram stain and other stains have been used after detecting a positive culture, as well as selective culture plates for testing antimicrobial resistance. Phenotypic identification methods as API 20E, allowing the identification of almost 100 taxa, were considered the “gold standard” until 1992. Currently, automated versions are able to reduce the time consumed to obtain results, such as Vitek^®^ (bioMérieux, Marcy l’Etoile, France) [9]. Another time-saving method is peptide nucleic acid fluorescence in-situ hybridization (PNA FISH), a technology to test blood smears made directly from positive blood culture bottles that reports results in 2.5 h [10]. Finally, it must be noted that the use of molecular-based methods is essential for the identification of some bacteria; e.g., identification of mycobacteria by the combined application of multiplex PCR, RFLP, and MAS-PCR enables diagnosis of *Mycobacterium tuberculosis* from sputum [11], and the combination of Fite–Faraco (FF) staining and PCR provides a rapid and definitive diagnosis of *Mycobacterium leprae* [12].

### 2.2. Antibody-Based Tests

Latex agglutination and immunofluorescence allow the relatively fast determination of pathogens in clinical samples. ELISA assays have been used in clinical settings for over 50 years to detect antigens from pathogens and for serological testing. Once the DNA microarray technique was available, the concept of multiplex and multiarray ELISA started to be developed, but relatively few tests have been validated for in vitro diagnostic (IVD) applications. This complex technology requires automation and algorithm-driven design to provide robust and accurate results in clinical diagnostic settings. Recently, the ultrasensitive ELISA has been proposed for disease diagnosis, due to its ability to quantitatively detect trace amounts of biomarkers [13]. For instance, diagnosis of active pulmonary *M. tuberculosis* can be carried out by the accurate detection of the secreted immunogenic protein MPT64, allowing proper treatment of the disease and further analysis of treatment efficacy [14].

### 2.3. Molecular Techniques

Molecular techniques have become more important over time, due to their high specificity and sensitivity, and are now indispensable in developed country laboratories. Tests such as FilmArray (bioMérieux) and GeneXpert (Cepheid) have integrated nucleic acid extraction and multiplex PCR into the test cartridge [15]. Traditional methods, like PCR and real-time PCR, are unable to detect pathogens that remain in the human body in a latent state of infection (like tuberculosis), due the low concentrations of nucleic acids present in clinical samples. Digital PCR exploits the PCR capacity of amplification from a single molecule, obtained by serial dilution of the sample, and permits quantification of the DNA template. Similarly, droplet digital PCR divides the PCR mix into smaller reactions running independently and counting the final positive reactions. Bio-Rad Laboratories (USA), Thermo Fisher Scientific (USA) and RainDance^®^ (Bio-Rad, USA) are now providing ddPCR machines for research laboratories and hospitals [16]. Unculturable microorganisms or isolated colonies can be tested by multilocus polymerase chain reaction (PCR) followed by electrospray ionization mass spectrometry (PCR/ESI-MS). Thus, the presence of pathogens in the clinical sample after amplification of housekeeping genes provides a signal for testing [17]. Based on this technology, IRIDICA, developed by Abbott Molecular, has diverse platforms for the detection and identification of 780 bacterial and *Candida* pathogens and the presence of four selected antibiotic resistance markers, 200 molds and yeasts and 130 viral species.

### 2.4. High-Throughput Sequencing (HTS)

This technology allows sequencing of thousands to billions of DNA fragments simultaneously and independently. Its application to clinical microbiology is a new approach to the detection of virtually any pathogen directly from clinical samples. Additionally, it offers the possibility of finding out other interesting information such as lineage tracing, strain identification and drug resistance, as well as quantitative or semiquantitative data regarding the concentration of microorganisms in the sample. In terms of diagnosis, it is especially useful when disease etiology is unclear or the list of potential pathogens is broad. Some examples of the identification of viruses [18] and bacteria [19] by this method have been reported. Platforms from Illumina, Thermo Fisher, and Pacific Biosciences require proper logistics and high investment as well as trained personnel. Oxford Nanopore Technologies (ONT) introduced the MinION, GridION, and PromethION platforms, which have a lower cost and the possibility of being used outside the laboratory in many different environments [20].

Remarkably, the development of isothermal amplification technologies has enabled the amplification of genetic material without repeated temperature cycles by using different enzymes. Other strategies are helicase-dependent isothermal DNA amplification, which utilizes a DNA helicase to generate single-stranded templates for primer hybridization and subsequent primer extension by a DNA polymerase [21], loop mediated isothermal amplification (LAMP) [22], nucleic acid sequence-based amplification (NASBA) [23], self-sustained sequence replication (3SR), and rolling circle amplification (RCA) [24].

However, a relatively long time to obtain results, together with the need for specialized personnel and expensive equipment have precluded the implementation of these techniques in low-resource settings. In the last two decades, there has been a trend to develop point-of-care testing (POCT), which is essential for the rapid detection and diagnosis of diseases, as well as for their monitoring.

### 2.5. Biosensors

Biosensor technology has attracted increased interest since it offers the specificity and sensitivity of biological systems integrated into small, low-cost devices. Electrochemical (amperometry, potentiometry, conductometry and impedometry) and optical (color, chemiluminescence or fluorescence, surface plasmon resonance (SPR) and surface-enhanced Raman spectroscopy) biosensors are the main techniques used so far [25]. Within medical use, electrochemical sensors are very useful and sophisticated tools for the detection of DNA/RNA from bacteria and viruses. In this type of sensor, nucleic acid hybridization and amplification produces a quantifiable electrochemical signal, which can be already detected with a very low amount of DNA/RNA present in the sample, thus making early detection of the disease feasible. Their importance lies in the fact that these devices are miniaturized, allow large-quantity manufacturing, and allow integration with other existing technologies [26]. In the current context of pandemic, where a global and decentralized response to diagnosis is needed, these devices are already being considered as an alternative and replacement to the standard PCR test. Optical biosensors represent the most common type and allow the sensitive and selective detection of a wide range of analytes including viruses, toxins, drugs, antibodies, tumour biomarkers and tumour cells. SRS technology is being widely used for the detection of bioaerosols that carry bacteria in the environment. This is very interesting for the control of airborne pathogenic bacteria, such as *Legionella*, *M. tuberculosis* and *Bacillus anthracis* [27]. This same technology is also useful for virus detection. For example, sensors based on entropy-driven strand displacement reactions (ESDRs) and double-layer DNA tetrahedrons (DDTs) are currently being used for HIV detection. Likewise, by applying Raman technology, it is possible to quickly and reliably detect the presence of viruses (such as the influenza virus) in human respiratory samples [28].

### 2.6. Lateral Flow Chromatography

Regarding the use of specific monoclonal antibodies, the development of rapid antigen detection tests for clinical samples has been an important step forward. This technology, known as lateral flow chromatography, provides near-patient information and results in approximately 15 min. Also, the lateral flow immunoassay can be carried out by antigen immobilization for testing the presence of specific antibodies in the patient. POC is playing an important role in resource-limited countries; however, the limited sensitivity of some POC tests concerns the WHO since false-negative outcomes might mean millions of lost lives [29]. This technology is extremely versatile and molecular techniques based on bacterial DNA detection can also be performed in a similar platform. After nucleic acid amplification, usually under isothermal conditions, the products can be run and visualized in lateral flow chromatography or microfluidic platforms.

### 2.7. Microfluidic Chips

Microfluidic chips are made of diverse materials, such as polymers, that combine micrometer-sized channels connected to the outside by inputs and outputs, in which liquids are injected by pressure systems. These devices also possess a detection system that could be combined with PCR, electrochemistry, or spectrometry, etc. One of their advantages is the decrease in detection time since microfluidic systems can integrate different steps, such as specimen preparation, reagent manipulation, bioreaction and detection, in a single platform [30]. When these devices integrate one or more laboratory functions they are known as lab-on-a-chip (LOC). An example is the detection of colistin-resistant bacteria by using loop-mediated isothermal amplification (LAMP) for DNA detection combined with semiconductor technology for non-optical real-time DNA sensing, and a smartphone application for data acquisition, visualization and cloud connectivity [31].

## 3. State-of-the-Art of New Therapies for Fighting Infectious Bacterial Diseases

The use of conventional antibiotics to treat bacterial infections should be just one strategy among many. Instead, it currently remains the only available option in most cases. Fortunately, several new approaches to fighting pathogenic bacteria are already in different stages of development. We can only hope that some of these strategies prove to be successful before bacterial infections become untreatable.

**Figure 2:**
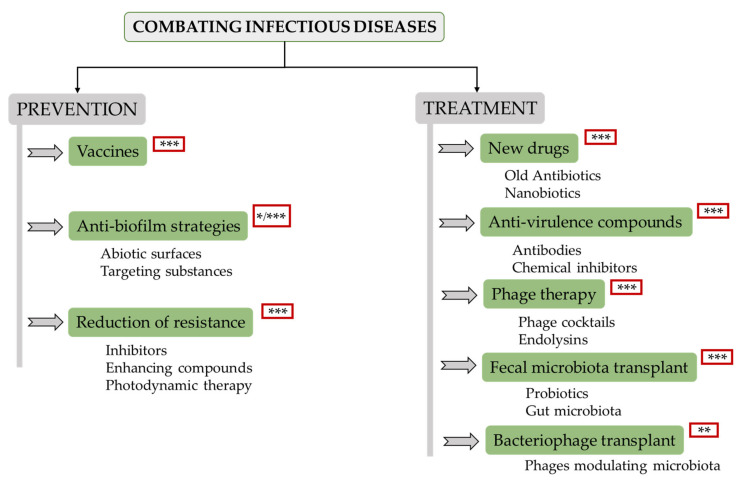
Overview scheme of potential alternatives to antibiotics that are currently under development. Level of development: * (low, laboratory research); ** (medium, animal models); *** (high, clinical trials).

Recent developments in this field allow the design of a complete tactic for prevention and treatment of these infections. Bacterial disease prevention remains a key challenge for various reasons. For instance, vaccines are not always easy to obtain, and reducing the contact with dangerous bacteria would be akin to creating a sterile environment, which would only be possible under specific conditions. Therefore, pathogenic microorganisms and their potential ability to develop drug resistance will always be a problem. With this in mind, there is ongoing research on various strategies to prevent and/or treat infectious diseases, the most important being the following (Figure 2):

### 3.1. Vaccines

Vaccination remains one of the most effective systems for infectious disease prevention. Additionally, the use of vaccines also contributes to mitigating the problems derived from infections caused by antibiotic-resistant bacteria. This is even the case for vaccinations against viral infections, as they result in a reduced frequency of superinfections with bacteria. A lesser prevalence of bacterial infections ultimately leads to a decrease in antibiotic prescription, thereby diminishing the selective pressure for antibiotic resistance. At the moment, vaccine development focuses on pathogens that represent a global threat to human health, antimicrobial-resistant ESKAPE (*E. faecium*, *S. aureus*, *Klebsiella pneumoniae*, *A. baumannii*, *P. aeruginosa*, and *Enterobacter* species) and pathogenic bacteria from the WHO priority list [32]. As a result of this effort, several vaccines against a number of pathogens are now in the pipeline (https://www.who.int/immunization/diseases/en/). Some examples of vaccines currently undergoing clinical trials include the *S. aureus* four-antigen vaccine, which appears to be well tolerated and induce high levels of antibodies [33], as well as vaccines against *A. baumanii*, *P. aeruginosa*, and *Streptococcus pneumoniae*. Still, new trends in vaccination include important advances in the design of glycoconjugate vaccines, which can be easily obtained by using *E. coli* cells to express both the saccharide antigen and the carrier protein, engineered outer membrane vesicles (OMVs) to simplify the purification of membrane antigens, and liposomes to improve their delivery [34]. Another promising area of vaccine development includes those that are based on DNA, with important progress to enhance their immunogenicity and delivery by electroporation and use of nanoparticles [35]. Finally, a new generation of vaccines is under development thanks to the use of new adjuvant molecules, particularly those that stimulate effective T-cell responses, such as RNA molecules that stimulate CD8 T-cell responses [36].

### 3.2. Antibiofilm Strategies

Biofilms are involved in multiple infections on both biotic (heart valves, tissues) and abiotic (catheters or prosthetic joints) surfaces. These structures are very difficult to eradicate because they exhibit multiple properties that hinder antibiotic activity. These include the physiological state of bacteria, the presence of persister cells, and the extracellular matrix. There are two routes of action to prevent biofilm formation: the development of abiotic surfaces and/or coatings that inhibit bacterial adherence to medical devices [37], and by targeting substances required for surface colonization, including adhesins, extracellular matrix components and regulatory signals [38,39]. Interference with regulatory signals may be achieved, for instance, by applying quorum sensing (QS) inhibitors. These compounds can act by preventing synthesis of the signaling molecules or by blocking the interactions between QS molecules and their receptors. Examples of QS inhibitors include apicidin, which inhibits the *S. aureus* agr system [40], and synthetic derivatives of natural furanone, which are potent antagonists of bacterial QS systems [41]. Biofilm formation can also be inhibited by using enzymes like glycoside hydrolases, α-amylase and cellulase, all of which degrade complex polysaccharides. There is evidence that these proteins are able to disrupt EPS from *S. aureus* and *P. aeruginosa* biofilms and increase the effectiveness of subsequent antibiotic treatments [42]. Unfortunately, these strategies are still in the research stage; therefore, further studies will be necessary prior to their clinical application. Another strategy to target the biofilm matrix involves the use of monoclonal antibodies directed against extracellular substances. For instance, AR-105 (Aerucin^®^) targets the alginate present in *P. aeruginosa* biofilms, which are often a problem in patients suffering from ventilator-associated pneumonia (VAP). AR-105 is currently being tested in phase II clinical trials (Aridis Pharmaceuticals Inc.).

### 3.3. Reduction of Antibiotic Resistance

A straight approach to treating infections caused by antibiotic resistant bacteria is the reduction of their ability to survive in the presence of antibiotics or under attack by the host’s immune system. One of these strategies is the use of compounds to specifically inhibit antibiotic resistance mechanisms, the classical example being the use of β-lactamase inhibitors in combination with a β-lactam antibiotic (e.g., amoxycillin and clavulanic acid) [43]. Similarly, it is possible to inhibit antibiotic resistance mediated by efflux pumps [44]. A second method to enhance antibiotic activity is by combining antibiotics with other substances. For instance, cyslabdan, a compound produced by *Streptomyces* sp. K04-0144, increases the activity of imipenem against methicillin-resistant *S. aureus* (MRSA) strains [45]. Finally, the use of photodynamic therapy (PDT) is an alternative strategy for destroying antibiotic resistant bacteria as effectively as susceptible cells by employing a non-toxic dye (photosensitizer) and low-intensity visible light. Illumination of the infected area in the presence of oxygen results in the production of cytotoxic molecules. In some cases, the photosensitizer increases the action of antibiotics. This is the case of the photoantimicrobial agent ATAZTMPo-gentamicin conjugate, which has activity against both Gram-positive and Gram-negative bacteria. Upon exposure to red light, this molecule enhances the activity of gentamicin and broadens its spectrum of action against pathogens [46]. A similar example is the use of this technology to inactivate the resistance mechanisms of bacteria. Thus, antimicrobial photodynamic therapy (aPDT) prevents carbapenem degradation, thereby enhancing the efficacy of this antibiotic against carbapenemase-producing pathogens [47]. Although the development of some such strategies is still in the preliminary phase, others have already reached the last steps towards their use in humans. For example, the application of indocyanine green-based photodynamic therapy against *Cutibacterium acnes* is undergoing clinical trials.

So far, we have described strategies aimed at limiting the onset of bacterial infections. However, other studies are tackling the development of novel therapies with improved efficacy against already existing infections. In this regard, the more important lines of research are the following:

### 3.4. New Drugs

Due to the difficulty of finding new molecules with antibiotic activity, an alternative is the use of old antibiotics, antibiotic combinations or even the application of compounds commonly used to treat other diseases [48]. The progress in high-throughput screening technologies and software development allows the identification of therapeutically interesting compounds by using in silico approaches (structure- (SBDD) and ligand-based drug design (LBDD), which has led to the development of several already approved therapeutics [49]. Additionally, a promising future is envisaged for nanobiotics, nanoparticles generated from a polymer-antibiotic conjugate, which releases the active drug upon hydrolysis in specific environments. For instance, some nanobiotics release their contents under acidic conditions such as those found within macrophages and granulomas [50]. Other types of nanoparticles (colloidal forms of silver, zinc, copper, titanium and vanadium) can also exert antimicrobial activity through the generation of reactive oxygen species, cell membrane permeation, triggering DNA damage or interrupting transmembrane electron transport [51].

### 3.5. Antivirulence Treatments

This strategy is based on the use of compounds targeting virulence factors produced by pathogenic bacteria. As a result, antivirulence compounds reduce pathogenicity and, consequently, contribute to bacterial clearance by the host’s immune system or antibiotic treatment. Common targets include toxins, adhesins, QS molecules, virulence-dedicated secretion and regulatory proteins, siderophores, and immune evasion factors. There are two main types of treatments, namely antibodies and chemical inhibitors. A nice example of toxin-directed antibodies is raxibacumab (GlaxoSmithKline), an already approved monoclonal antibody used to treat inhalational anthrax, a form of this infectious disease caused by breathing in the spores of the bacterium *B. anthracis* [52]. Another example is the FDA- and EMA-approved human monoclonal antibody Bezlotoxumab (Merck), which blocks *C. difficile* toxin B and prevents recurrent infections by this pathogen [53]. Similar antibodies are being assayed to target toxins secreted by other pathogens such as *S. aureus.* Chemical inhibitors are usually directed against toxin secretion systems to inhibit their activity [54]. ZnO nanoparticles have an inhibitory effect on QS-regulated genes controlling virulence factor secretion [55]. Monoclonal antibodies can also be directed against these targets. For instance, a monoclonal antibody (MEDI3902, Medimmune) targets the *P. aeruginosa* T3SS needle tip protein to prevent injection of toxins into the host cells and, at the same time, the Psl exopolysaccharide, which is involved in bacterial colonization, thus inhibiting attachment to epithelial cells [56]. The use of MEDI3902 to prevent VAP caused by *P. aeruginosa* is currently undergoing phase II clinical trials. A further example is the monoclonal antibody mAb 514G3, able to neutralize *S. aureus* protein A (SpA), whose effectiveness for the treatment of staphylococcal bloodstream infections is the focus of a phase I/II clinical study (XBiotech NCT02357966) [57]. A different strategy is to favor pathogen removal by the host immune system or improve the action of antibiotics. An example is the high-density lipoprotein (HDL) that binds to streptococcal collagen-like protein enhancing phagocytosis [58]. Other non-traditional approaches include the use of immunomodulating agents such as Reltecimod, a peptide that reduces acute inflammation and protects from superantigen toxins. This therapy is in phase III clinical trials (AtoxBio) for the treatment patients with necrotizing soft-tissue infections in addition to the standard treatment (https://www.globenewswire.com/news-release/2020/07/10/2060386/0/en/Atox-Bio-Announces-a-Positive-Effect-of-Reltecimod-on-Resolution-of-Organ-Dysfunction-in-Phase-3-ACCUTE-Trial-for-Patients-with-Necrotizing-Soft-Tissue-Infection-Flesh-Eating-Disea.html).

### 3.6. Phage Therapy

Bacteriophages can theoretically be used as therapeutics against any bacterial infection. Recently, phage therapy has attracted a lot of attention due to the successful treatment of a modest number of patients under compassionate use programs by administration of personalized phage preparations [59]. So far, several publications have reported the use of phage therapy against a wide variety of infectious diseases, including gastrointestinal infections, burns, chronic venous ulcers, as well as systemic, urogenital, respiratory, otorhinolaryngeal, and osteoarticular infections [60,61]. In view of this increasing number of successful cases, several companies are putting effort and investment into the development of phage cocktails; e.g., Pherecydes Pharma is working on a therapy for osteomyelitis and prosthesis infections. Moreover, phage-derived proteins (endolysins) are also gaining attention because of their efficient bacteriolytic activity. Endolysins are being developed as a pharmaceutical product for application against human and animal pathogens (companies ContraFect, Intron Biotechnology, GangaGen and Micreos are conducting phase II clinical trials), as well as in the cosmetic industry (the GladSkin brand with Staphefekt ™ against *S. aureus* commercialized by Micreos). Recent clinical trials in humans (phase I) have been conducted with two endolysins: iNtRON-N-Rephasin^®^ SAL200 (Tonabacase) (iNtRON Biotecnology, 2020) and Exebacase (CF-301) (ContraFect Corporation, 2019) against *S. aureus* bacteremia without observing any adverse effect. These two proteins are currently undergoing phase II and phase III trials, respectively. Although phages have been used historically in infection treatment, important challenges and scientific questions (phage specificity, pharmacokinetics, resistance development, or potential virulence transfer) should be solved before translating this therapy to routine clinical practice. Nonetheless, the future of phage therapy is promising because there are new possibilities of adapting these antimicrobials by using genetic tools such as CRISPR–Cas systems [62]. Similarly, extensive protein engineering will allow the use of endolysins against Gram-negative bacteria [63].

### 3.7. Faecal Microbiota Transplant

Nowadays, the importance of the microbiota in human health has been well established. In fact, a significant number of examples show a correlation between some diseases (diabetes, cardiovascular disease, mental health) and the microbiota state. Moreover, the use of probiotics and fecal microbiota transplantation have been shown to reduce the incidence and severity of some bacterial infections, especially those more difficult to eradicate or caused by antibiotic resistant bacteria, such as *Clostridium difficile* [64]. Furthermore, synthetic biology allows the specific genetic engineering of probiotics [65]. Despite promising results from controlled trials, the efficacy of probiotics for disease prevention has not been fully demonstrated yet. In contrast, fecal microbiota transplants have been shown to restore the gut microbiota in patients and significantly reverse the predisposition for colonization with multiresistant pathogens [66].

### 3.8. Bacteriophage Transplant

Bacteriophages are in dynamic interaction with microorganisms colonizing the human gut; therefore, it is theoretically possible to use them for modulating the microbiota. This effect can be achieved by performing bacteriophage transplants. Research on this topic is still in the first stages, but experiments carried out in animal models have found that phage predation impacts on the number of susceptible bacteria and, indirectly, on other bacterial species, which has noticeable consequences for the gut metabolome [67]. Moreover, there is evidence about the persistence of bacteriophages and their impact on long-term microbial dynamics in human beings after fecal microbiota transplantation [68].

## 4. Future Challenges

Given the importance of diagnostic systems to contain the spread and reduce the damage of infectious diseases at a sufficiently early stage, the WHO has provided the “ASSURED” definition of the ideal diagnostic test (Affordable, Sensitive, Specific, User-friendly, Rapid, Equipment-free and Delivered to those who need it). However, the development of numerous tests in the last years has been devoid of studies assessing their performance in real settings, especially in low-resource environments, as well as regulatory systems [69]. One of the problems associated with test development is the selection of the type of clinical sample that should be used for detection. Considering that diagnostic tests might be used by unqualified personnel, samples should also be easy to obtain and process, and stable during storage. Another drawback of most molecular methods is the need to previously obtain genetic material, as this step is usually carried out manually. Regarding sample processing, it would be desirable that the POC includes all steps necessary to obtain results, especially in biosafety restricted conditions and when the samples are highly infectious. Still, easy handling and low cost are essential characteristics so that any diagnostic test can be implemented in countries all over the world. Concerning new therapies, there is a trend towards narrow-spectrum or pathogen-specific drugs, a strategy that will also require a highly developed diagnostic infrastructure. Moreover, the approval and commercialization of these drugs is more difficult, among other reasons, because clinical trials require screening of a large number of patients. Regarding costs, personalized medicine implies a higher cost per patient, making it less accessible in developing countries. In this context, prevention of infectious diseases by vaccination plays an important role in reducing the incidence of some infections in these countries. In this context, affordable treatment strategies, like phage therapy, might prove to be a good choice since phage products can be developed faster and at a lower cost than conventional drugs.

## 5. Conclusions

The progress in human medicine, specifically in combating infections, derived from the discovery of antibiotics in the twentieth century has been compromised by the spread of antibiotic resistance in the present century. To overcome this drawback, the engagement of several disciplines is of paramount importance. Thus, environmental and epidemiological studies are relevant to design containment mechanisms; veterinary science to prevent animals from being vehicles and reservoirs of resistant pathogens; public health for a proper management of surveillance and control of resistance development; medicine, immunology and pharmacology are necessary for the rational design of new therapies; and, last but not least, molecular biology and chemistry will help to advance our understanding of disease etiology and pathogenesis. The acquisition of basic knowledge from omics (genomic, transcriptomic, proteomic and metabolomics) data is contributing to drug development and design of treatments against most pathogens. Beyond that, addressing the global control and spread of some diseases will require a global socio-political effort. Thus, the role of global organizations and governments will be crucial towards infectious disease control. Despite the significant advances made, infectious diseases represent a continuous challenge due to the emergence of new and/or more virulent pathogens and the change in other factors (social, political, environmental, technological and environmental) that favor their spread. This fact requires constant surveillance and intervention as well as proper technologies to support diagnosis and treatment.
